# Co-Culture of Tumor Spheroids and Fibroblasts in a Collagen Matrix-Incorporated Microfluidic Chip Mimics Reciprocal Activation in Solid Tumor Microenvironment

**DOI:** 10.1371/journal.pone.0159013

**Published:** 2016-07-08

**Authors:** Su-Yeong Jeong, Ji-Hyun Lee, Yoojin Shin, Seok Chung, Hyo-Jeong Kuh

**Affiliations:** 1 Department of Biomedicine & Health Sciences, Graduate School, The Catholic University of Korea, Seoul, Republic of Korea; 2 Department of Mechanical Engineering, Massachusetts Institute of Technology, Cambridge, Massachusetts, United States of America; 3 School of Mechanical Engineering, Korea University, Seoul, Republic of Korea; 4 Cancer Evolution Research Center, College of Medicine, The Catholic University of Korea, Seoul, Republic of Korea; 5 Department of Medical Life Sciences, College of Medicine, The Catholic University of Korea, Seoul, Republic of Korea; Seoul National University, REPUBLIC OF KOREA

## Abstract

Multicellular 3D culture and interaction with stromal components are considered essential elements in establishing a ‘more clinically relevant’ tumor model. Matrix-embedded 3D cultures using a microfluidic chip platform can recapitulate the microscale interaction within tumor microenvironments. As a major component of tumor microenvironment, cancer-associated fibroblasts (CAFs) play a role in cancer progression and drug resistance. Here, we present a microfluidic chip-based tumor tissue culture model that integrates 3D tumor spheroids (TSs) with CAF in proximity within a hydrogel scaffold. HT-29 human colorectal carcinoma cells grew into 3D TSs and the growth was stimulated when co-cultured with fibroblasts as shown by 1.5-folds increase of % changes in diameter over 5 days. TS cultured for 6 days showed a reduced expression of Ki-67 along with increased expression of fibronectin when co-cultured with fibroblasts compared to mono-cultured TSs. Fibroblasts were activated under co-culture conditions, as demonstrated by increases in α-SMA expression and migratory activity. When exposed to paclitaxel, a survival advantage was observed in TSs co-cultured with activated fibroblasts. Overall, we demonstrated the reciprocal interaction between TSs and fibroblasts in our 7-channel microfluidic chip. The co-culture of 3D TS-CAF in a collagen matrix-incorporated microfluidic chip may be useful to study the tumor microenvironment and for evaluation of drug screening and evaluation.

## Introduction

Preclinical cancer models with high clinical relevancy are essential not only for efficient drug screening during early drug development but also for studies of pharmacological mechanisms of drugs or drug targets under clinical development or investigation [1]. It is widely accepted that culturing cells in two-dimensional (2D) condition is not physiologically relevant, and translation into *in vivo* may not be successful [2]. Tissue-specific architecture, based in part on interactions with microenvironmental elements, is an essential component of a tumor and may be recapitulated in three-dimensional (3D) cell culture models [2]. Tissue-like structures and properties of 3D cell models are conferred by providing and facilitating cell-cell and cell-extracellular matrix (ECM) interactions. Hence, 3D cell cultures are considered highly predictive models. Examples of 3D culture models commonly used include multicellular layer models, which reflect certain aspects of solid tumor tissues with intermediate complexity [3]. Additionally, tumor microtissues or tumor spheroids (TS) are widely used as 3D models representing avascular tumor regions. For multilayer tissue models, 3D cell cultures using hydrogels [4], polymer scaffolds [5], microcarrier beads [6], and hanging droplets [7] have been developed. These technologies have been exploited to study tumor-specific phenomena including drug transport and binding [8], chemo-resistance and cell invasion [9]. The transition to 3D cell culture models is critical by which better biomimetic tissue models can be accomplished [10].

The major role of the tumor microenvironment in cancer progression, metastasis and drug resistance has gained increased attention [11,12]. Recently, there have also been several studies on stroma-mediated drug resistance in tumors [13,14]. Context of tumor microenvironments is variable depending on the tissue origin and progression stages. They generally consist of tumor vasculature, an extracellular matrix, cancer-associated fibroblasts, and activated immune cells that all interact with cancer cells via not only paracrine signaling but also cell-to-cell contact mechanisms [15]. Allowing for interaction with components of the surrounding microenvironment is considered essential to establishing a ‘more clinically relevant’ tumor model. Simulating these interactions between two or more pertinent cell types through co-culture can improve the overall biological relevance of a cell culture model as several studies have reported co-culturing different types of cells [16]. Over the past several years, various types of co-culture systems implementing this mutual interaction have gained attention in the cancer research field. 3D cultures embedded in a matrix are increasingly utilized to study the processes and mechanisms of differentiation, invasion, and migration of tumor cells induced by extracellular matrix components [17]. These methods also have been used to test anticancer agents, for example, an EMT-blocking agent tested in co-culture model between lung carcinoma cells and human umbilical vein endothelial cells (HUVEC) [18].

Among the cellular interaction within the tumor microenvironment, the interaction between cancer cells and fibroblasts is known to contribute to tumor initiation, progression and metastasis in many cancer types [19]. Cancer-associated myofibroblasts are often increased in numbers and heterogeneous as reported in colorectal carcinomas [20]. Paracrine signaling between fibroblasts and carcinoma cells have shown a mutual stimulation of proliferation and induction of drug resistance [13].

Microfluidic technology was introduced recently into the biological sciences field, such as organ-on-a-chip system. Microfluidic techniques are useful in controlling spatial arrangement of cell growth and fluids within micrometer-sized channels, which may be exploited to increase the physiological relevance of tumor models [10, 21]. They can provide an advantage in mimicking the microscale interactions within tumor microenvironments, and may replace the conventional use of conditioned medium or conventional culture devices that employ non-physiologic distances between interacting compartments. Cell culture in microfluidic systems often used hydrogels for cell embedment or encapsulation to provide 3D architecture and composition [22–25]. Formation of perfusable 3D microvessel *in vitro* [25] as well as cancer metastasis such as extravasation and micrometastasis generation [24] are the popular research areas where the microfluidic platform has been widely utilized. This culture platform has been extended to co-culturing cancer cells with fibroblasts [15]. We also exploited this technology to mimic a microenvironmental condition that recapitulates heterotypic interactions among cancer cells within 3D tumor spheroids and between cancer cells and stromal fibroblasts and stromal matrix in 3D context.

The combination of 3D co-culture with microfluidic technology offers a great potential for *in vivo* tumor-like model system and extend applications to the 3D co-culture based drug screening and mechanism studies [26]. In this study, we established a 3D co-culture model for human colorectal tumor using microfluidic chip. Tumor spheroids grown within collagen-incorporated microchannel were co-cultured with colorectal fibroblasts in a microscale distance away, allowing reciprocal activation as in *in vivo* tumor microenvironment. In our model, we observed *in vivo*-like characteristics strongly supporting its usefulness as a preclinical tumor model for drug screening and for the study of tumor microenvironmental interactions.

## Materials and Methods

### Cell culture

Human colorectal cancer cell line HT-29 was purchased from the Korean Cell line Bank (Seoul, Korea). Cells were maintained in RPMI1640 (Gibco BRL, Grand Island, NY) supplemented with 100 μg/mL streptomycin, 100 units/mL penicillin, 250 ng/mL amphotericin B and 10% fetal bovine serum (FBS, Welgene, Daegu, Korea) in a humidified atmosphere (5% CO_2_ / 95% air) at 37°C. CCD-18Co human normal fibroblast cell line was obtained from the American Type Tissue Culture Collection (ATCC) and cultured in MEM/EBSS (Hyclone, Logan, UT) supplemented with 2 mM sodium bicarbonate, 1 mM sodium pyruvate (Hyclone), non-essential amino acid (Sigma-Aldrich, St. Louis, MO) and 10% heat-inactivated fetal bovine serum.

### Fabrication of PDMS microfluidic chip

Microfluidic chips were made using poly-dimethylsiloxane (PDMS; Silgard 184, Dow Chemical, Midland, MI). A SU-8 patterned master was prepared using photolithography and then a conventional soft lithography was used on the SU-8 patterned master to produce PDMS replicas as previously reported [27]. In brief, PDMS base and curing agent were mixed thoroughly at a ratio of 10:1 (w/w) and poured over the SU-8 master and cured for 3 hr at 60°C. Inlet and outlet ports were made for loading/withdrawal of cell-hydrogel mixture and media using 18G needle and 6 mm disposable biopsy punch, respectively. Open side of PDMS replicas was bonded to a glass coverslip or a film of PDMS membrane (~80 μm thick; Amed Co., Seoul, Korea) with oxygen plasma (Femto Science, Seoul, Korea) for channel formation. Microfluidic channels were then coated with poly-dopamine solution (2 mg/mL) to promote type I collagen adhesion onto the channel surface as previously reported [28–29]. Chips were dried overnight at 60°C in an oven and used for experiments within 3 days.

### Culture of cell-hydrogel mixture in microfluidic channels

Cells were harvested and cell suspension was prepared at 5×10^6^ /mL for HT-29 and 3×10^6^ /mL for CCD-18Co. Collagen gel solution (2 mg/mL) was prepared by mixing collagen type I (rat tail, BD Biosciences, San Jose, CA) with phenol red-containing PBS with pH adjusted to 7.4 using 0.5 N NaOH. The cell suspension was mixed with the type I collagen solution at a 1:9 ratio and 3.5×10^3^ of HT-29 cells (into one channel) and 4.2×10^3^ of CCD-18Co cells (divided into two channels) were loaded into each designated channel by injecting 7 μL of cell-hydrogel mixture into the gel channels. We used a fixed ratio of cancer cells to fibroblasts at 1:1.2 which was in the range reported for *in vivo* relevancy. The carcinoma-stromal ratio has been reported to vary among patients (20% ~ 90%) [30] and the ratios of cancer cells to fibroblasts as 1:1 to 1:3 have been usually studied in many *in vitro* studies [31–32].

After polymerization in a humidified 5% CO_2_ incubator at 37°C for 30 min, microchannels were filled with culture medium, and returned back to the incubator for culture. Medium change was done every day and cells were cultured for 5 days. Diameter of spheroids was calculated using bright field images and the area obtained from image J assuming a circular shape of spheroids based on an equation (area = π*r*^2^. Cell aggregates of diameter larger than 50 μm were considered as spheroids.

### Immunofluorescence staining and imaging

Expression of Ki-67, fibronectin, α-SMA and F-actin was detected using immunofluorescence staining in day 6. Briefly, cells were fixed in 4% paraformaldehyde for 30 min and treated with 0.5% Triton X-100 for another 30 min. After blocking non-specific binding with 5% bovine serum albumin (BSA, Affymetrix, Cleveland, Ohio) for overnight, primary antibodies against fibronectin (1:50, ab2413, Abcam, Cambridge, UK), Ki-67 (1:50, sc-15402, Santa Cruz, Dallas, TX), α-SMA (1:50, ab5694, Abcam), F-actin (1:50, cat. No. R415, Thermo Fisher, Waltham, MA) were applied at 4°C overnight. After incubation with secondary antibody (1:2000, cat. no. Z-25307, Thermo Fisher) and DAPI (1:1000, cat. No. D9564, Sigma-Aldrich) at room temperature for 3 hr. Microchannels were washed with PBS and subjected to confocal microscopy (LSM 510 Meta, Zeiss, Oberkochen, Germany). Optical sections were acquired at 3 μm intervals and stacked into a z-projection from which fluorescence intensity was calculated. For quantitative comparison, data were normalized to DAPI intensity.

### Drug uptake and response assay

Doxorubicin (DOX) was used to evaluate drug accumulation in spheroids because of its fluorescence property. Sensitivity to paclitaxel was tested: drug selection was based on a current clinical trial investigating an efficacy of Abraxane® (Nab-Paclitaxel) in metastatic colorectal cancer [33]. After 5 day culture, media was replaced with drug containing media. After 2 hr, cultures were washed with PBS before imaging to remove background fluorescence (confocal microscopy). DOX intensity was calculated as an average of three representative fields out of 8 total fields covering the effective area in each channel. Optical sections were acquired at 3 μm intervals and stacked into a z-projection from which fluorescence intensity was calculated.

For viability assay, 5 day-cultures of spheroids and fibroblasts were exposed up to 300 μM of paclitaxel for 72 hr and stained using LIVE/DEAD reagents (BDA-1000, BIOMAX, Seoul, Korea) according to the procedure provided by manufacturer. Cultures were washed with PBS before imaging to remove background fluorescence (confocal microscopy). Optical sections were acquired at 3 μm intervals and stacked into a z-projection. Images were quantified using image J software as described above.

### Human proteome array analysis

The expression levels of proteins known for their roles in angiogenesis, apoptosis and cell motility were analyzed using the Proteome Profiler™ (Human Angiogenesis Array kit, Human Apoptosis Antibody Array kit, R&D System, MN) according to the manufacturer's instruction. The 28 antibodies of angiogenesis array kit were used in the experiment (S3 Fig). Briefly, cell lysates were prepared from cells cultured in PDMS film-bonded PDMS chips for 6 days. After blocking of non-specific binding at room temperature for 1 hr, antibody array membranes were incubated with the cell lysate (1.5 mL) at 4°C overnight and then with a diluted solution of horseradish peroxidase-conjugated streptavidin at room temperature for 30 min. Visualization was done by chemiluminescence and signal intensity was quantified using Multi Gauge V3.0 software (FUJI FILM, Japan).

### Statistical analysis

All data were expressed as the mean±standard deviation (SD) of three or more independent measurements. Student t-test as well as χ^2^ test were used to test the statistical significance using Microsoft Excel 2010. P values < 0.05 were considered statistically significant.

## Results

### 3D culture of tumor spheroids and fibroblasts in microfluidic chip

**Fig 1:**
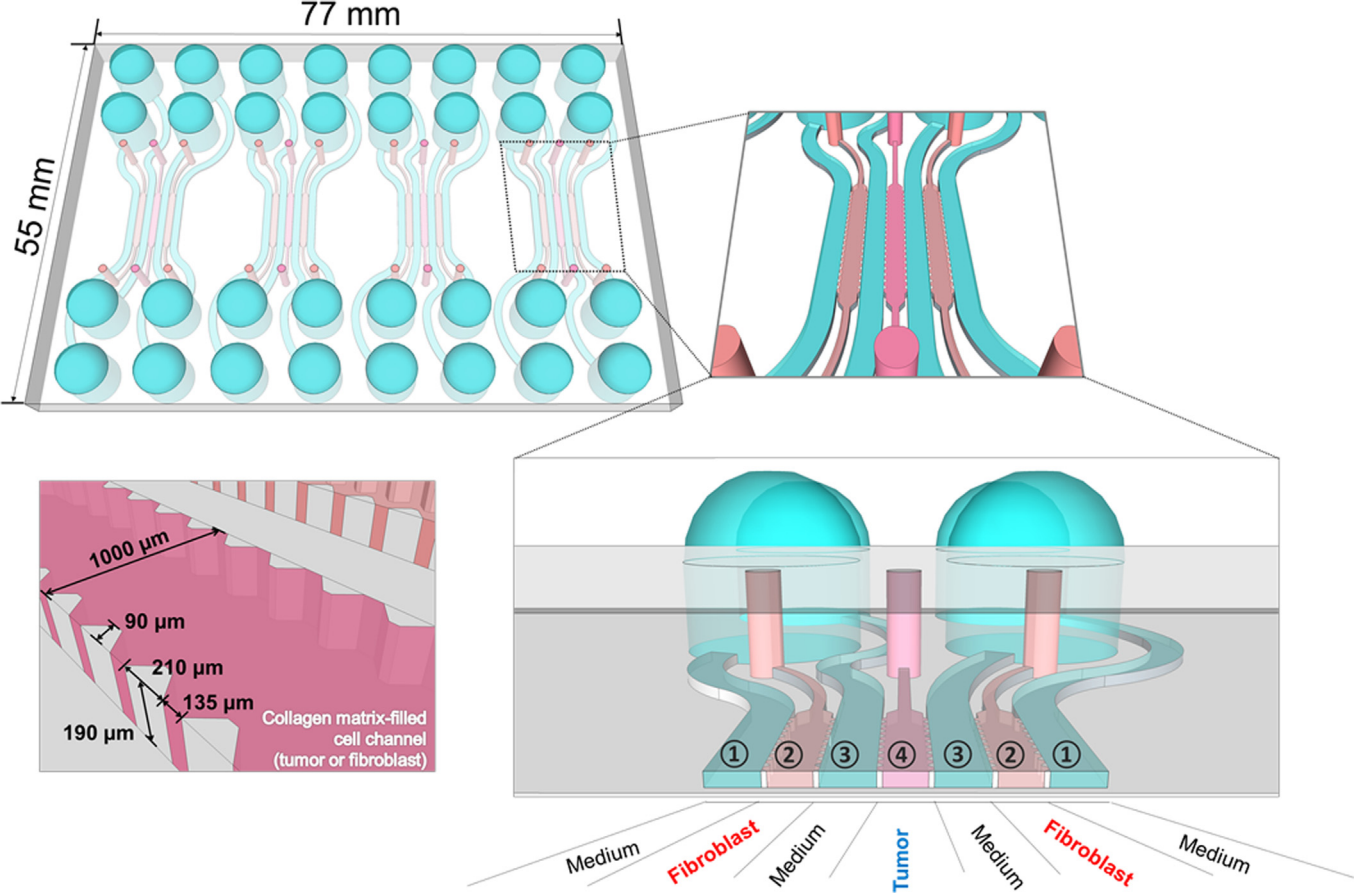
Design of microfluidic chip for tumor spheroid-fibroblast co-culture. Structure and organization of a microfluidic chip used for 3D co-culture of human colorectal cancer cells (HT-29) and normal colorectal fibroblasts (CCD-18Co). One chip contained 4 units and one unit consisted of 7 channels for either cell loading or media fill. Channel designation for co-culture: cancer cells and fibroblast cells were loaded in channel 4 and 2, respectively, and other channels (1 and 3) were used for media fill. A cell loading channel is shown with detailed structure and dimension (left-bottom).

We designed a microfluidic chip to study 3D interactions between cancer cells and fibroblasts that reflect the tumor microenvironment *in vivo*. Fig 1 represents a schematic of the chip design used in this study. Each chip contains four units and each unit consists of three cell channels and four medium channels. Channel width was 1000 μm and channel depth approximately 190 μm, and material/gas exchange was accommodated between channels. For co-culture, type I collagen suspension of HT-29 cells and normal colon fibroblasts (CCD-18Co) was loaded in the center channel (channel 4 in Fig 1) and the side channels (channel 2 in Fig 1), respectively. In mono-culture, either HT-29 or CCD-18Co was mixed with type I collagen solution and loaded in the center channel only and type I collagen solution without cells was loaded in the others two channels.

**Fig 2:**
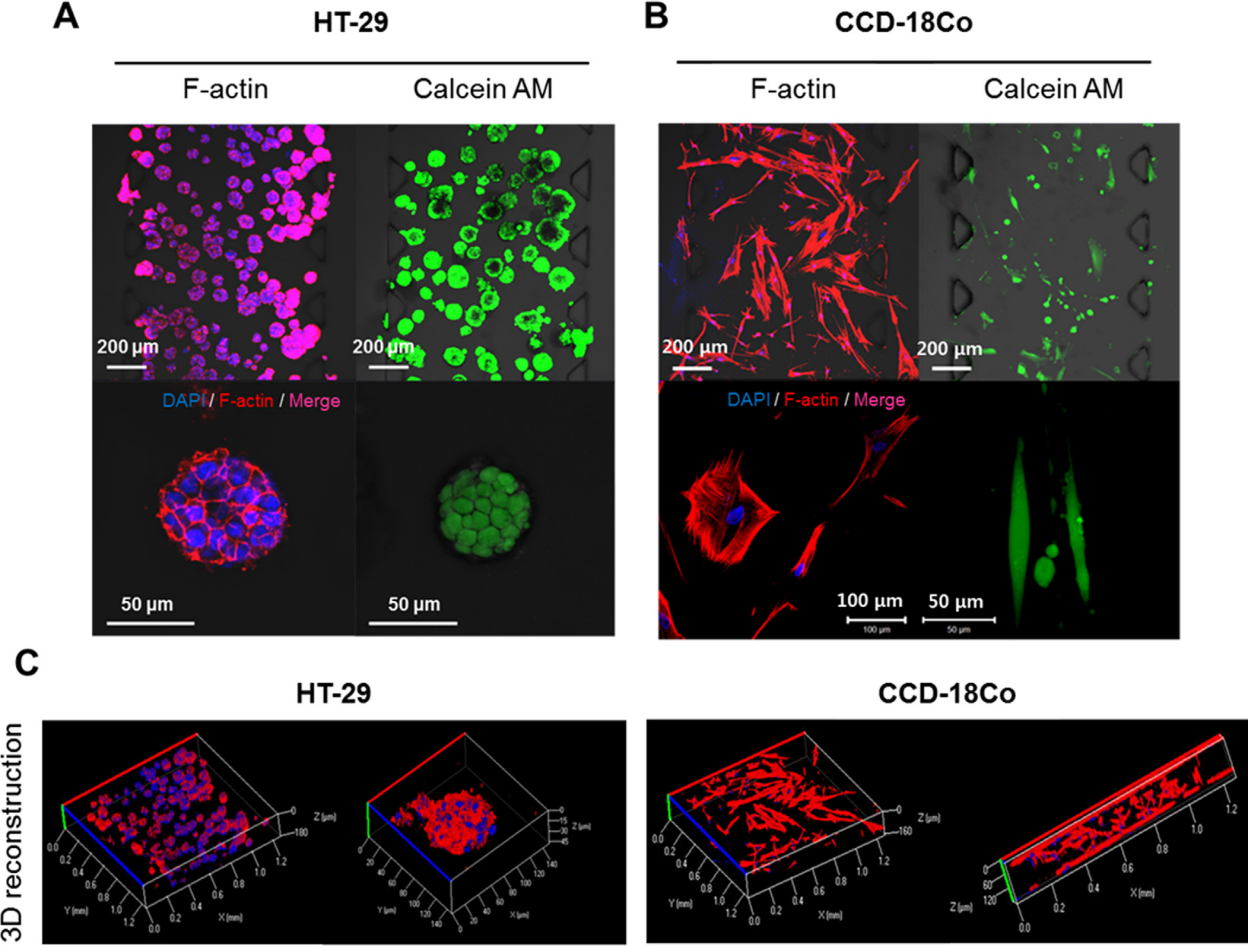
3D Culture of tumor spheroids and fibroblasts in 7-channel microfluidic chip. Fluorescence images of HT-29 tumor spheroids (A) and CCD-18Co (B). Cells were stained for nuclei (DAPI) and F-actin (left panel) or for viability with calcein AM (right panel); (scale bar = 50 μm to 20 μm as indicated in each picture). (C) 3D reconstruction images of HT-29 tumor spheroids and CCD-18Co fibroblasts cultured within channels. Both cells were grown for 5 days in collagen matrix-supported microfluidic channels to form 3D spheroids cultures. Optical sections were acquired at 3 μm intervals and stacked into a z-projection or 3D reconstruction images.

After 5 days of culture in these collagen-incorporated microfluidic channels, HT-29 cells formed viable 3D spheroids as shown by fluorescence staining of F-actin and calcein AM (Fig 2A). The morphology and viability of CCD-18Co fibroblasts were also confirmed by the same staining (Fig 2B). The growth of both cells in the microfluidic chip resulted in 3D expansion as confirmed with reconstructed images following optical sectioning using confocal microscopy (Fig 2C). HT-29 spheroids and CCD-18Co cells proliferated within the space of the corresponding channels over 5 days, during which their growth and interaction was monitored and characterized.

### Effect of fibroblast co-culture on the growth of HT-29 tumor spheroids

**Fig 3:**
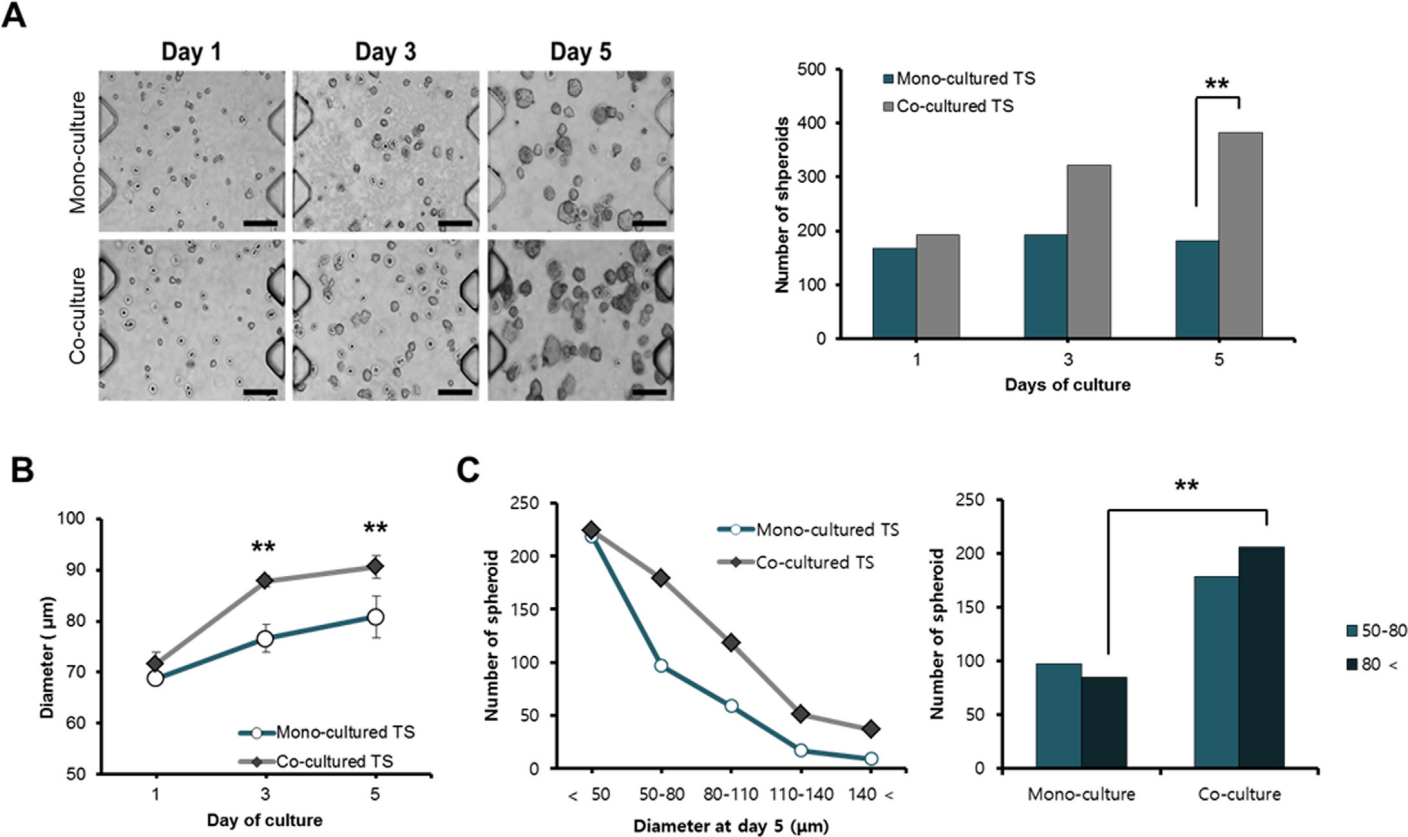
Effect of co-culture on growth and size distribution of spheroids. Cells were grown in collagen-supported microfluidic channels with or without fibroblast co-cultures and number and size distribution was determined at day 5. (A) Growth of HT-29 spheroids in size and number (scale bar = 200 μm). (B) Comparison of mean diameter of HT-29 spheroids over 5 days. (C) Comparison of size distribution of tumor spheroids on day 5. Diameter of spheroids was calculated using bright field images and Image J program. Cell aggregates of diameter larger than 50 μm were considered as spheroids. Student t-test as well as χ^2^ test were used for the statistical significance. Data are expressed as the mean ± SE of 3 replicates. * p<0.05, **p<0.01.

HT-29 cells were cultured as 3D TS in the collagen-matrix supported channels and monitored for their growth over 5 days under either mono- or co-culture conditions with CCD-18Co fibroblasts. Significant difference was observed in size but not in number of TS formed between mono- and co-culture conditions until day 3 (Fig 3A and 3B). By day 5, co-cultured spheroids showed an apparent significant increase in size and number as compared to mono-cultured spheroids (Fig 3A and 3B). A spheroid growth-promoting effect from fibroblast co-culture was prominent when the average size of TS was compared between mono- and co-cultured TS, i.e., average diameter of TS showed a 26% increase from day 1 to day 5 when co-cultured with fibroblasts whereas mono-cultured spheroids only showed a 17% increase (Fig 3B). When mono-cultured, more than 55% of spheroids (219 out of 401) were found to be smaller than 50 μm and only 20% (85 out of 401) were characterized as ‘large’ spheroids (with diameter over 80 μm) (Fig 3C). In contrast, ‘small’ spheroids (under 50 μm) composed only 36% (224 out of 609) and a significantly higher fraction (33%, 206 out of 609) appeared as ‘large’ spheroids under co-culture conditions (Fig 3C). It was noteworthy that increased number and diameter of co-cultured TS reflected increased cell proliferation compared with mono-cultured TS. Number and diameter of spheroids were calculated as an average of three representative image fields out of 8 total fields covering the effective area in a channel, except size distribution which was obtained from total fields from a channel.

### Effect of fibroblast co-culture on ECM expression and drug uptake of HT-29 tumor spheroids

**Fig 4:**
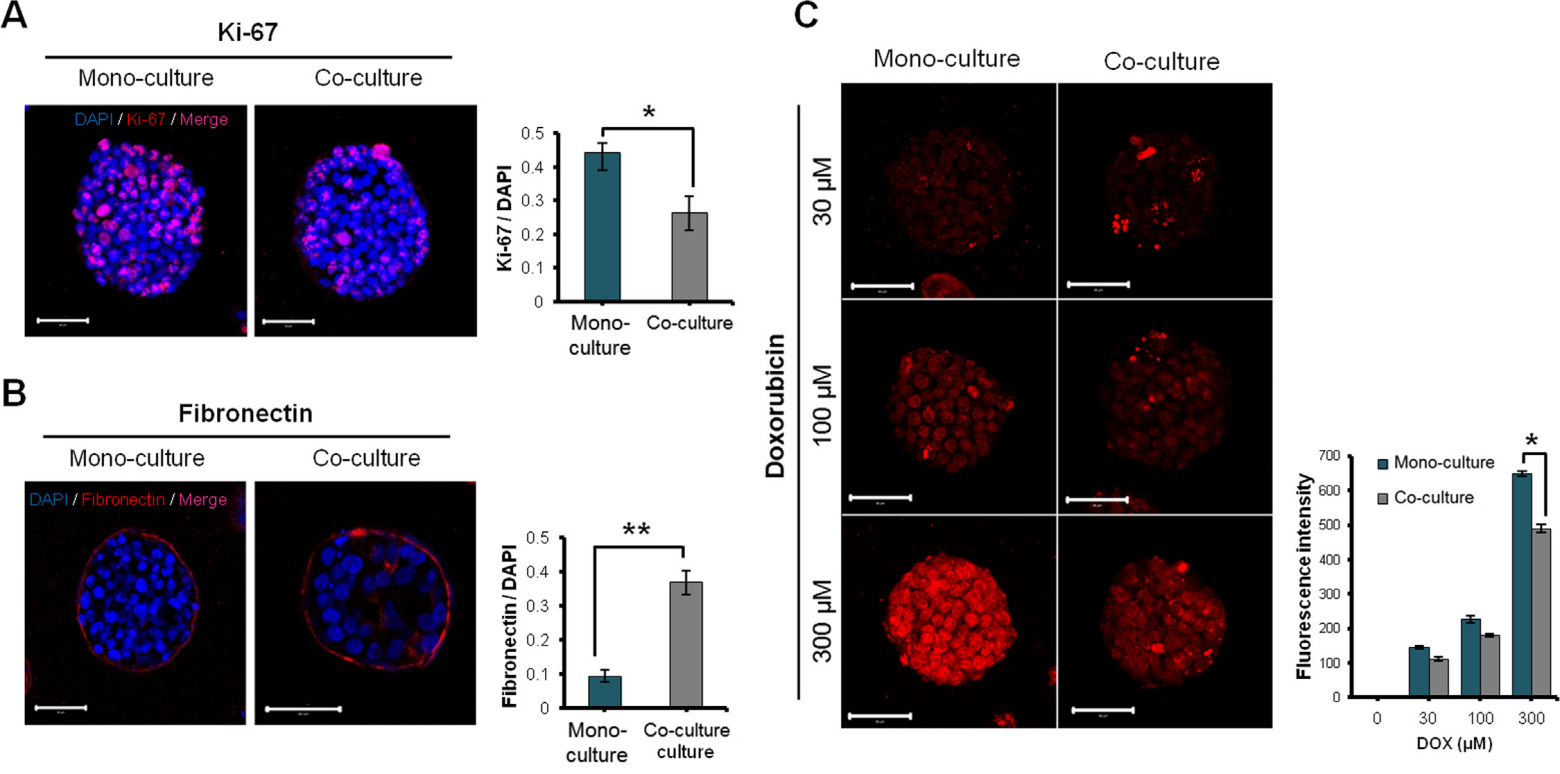
Effect of co-culture with fibroblast on proliferation, ECM expression, drug penetration in HT-29 tumor spheroids. (A-B) Effect of fibroblast co-culture on the expression of Ki-67 and fibronectin. (C) Differential uptake of DOX in tumor spheroids with or without fibroblast co-cultures after 2 hr exposure. HT-29 cells were grown in collagen matrix-supported microfluidic channels to form 3D spheroids. Spheroids cultured for 6 days were subjected to immunofluorescence detection of Ki-67 and fibronectin. Optical sections were acquired at 3 μm intervals and stacked into a z-projection from which fluorescence intensity was calculated. Student t-test was used to test the statistical significance. Data are expressed as the mean ± SE of 3 replicates. (Scale bar: 50 μm). * p<0.05, ** p<0.01.

Despite the apparently greater growth rate (Fig 3B), the fraction of cells expressing the proliferation marker Ki-67 was lower in the spheroids co-cultured with fibroblasts (Fig 4A). Fibronectin expression in HT-29 TS grown in type I collagen matrix either with or without fibroblasts was mainly observed in the peripheral region of the spheroids. When co-cultured with fibroblasts, significantly higher levels of expression were measured due to increased expression in the inner region of spheroids (Fig 4B). Penetration of DOX into TS was complete within 2 hr and the uptake increased in a concentration-dependent manner in the both spheroids with or without fibroblasts (Fig 4C). In particular, significantly lower levels of DOX uptake were observed in the co-cultured spheroids, which may be related to the increased levels of fibronectin (Fig 4B).

### Morphological changes and activation of fibroblasts under co-culture condition with 3D tumor spheroids

**Fig 5:**
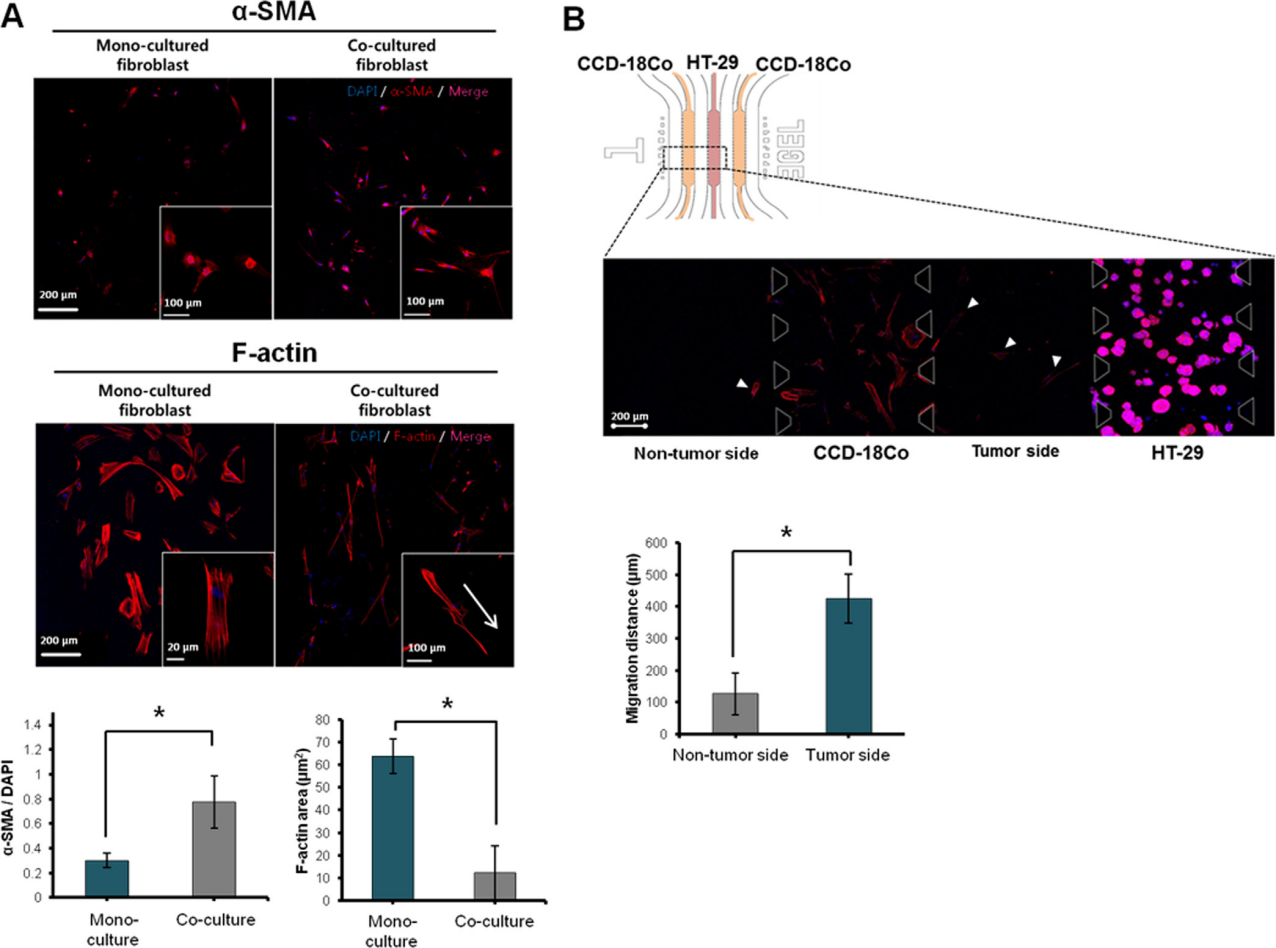
Activation of fibroblasts under co-culture with 3D tumor spheroids. (A) Fluorescence images of fibroblasts stained for F-actin and α-SMA showing differential expression levels under mono- and co-culture conditions. (B) Increased migration ability of fibroblasts towards 3D tumor compartment. A picture for a representative regions showing fibroblast migrated out of the designated channel and comparison of migration distance. The migration distance of fibroblasts was measured between the nucleus of fibroblast in medium channel and end of fibroblast culture channel. Cells were grown for 6 days before all measurements. Optical sections were acquired at 3 μm intervals and stacked into a z-projection from which fluorescence intensity was calculated. Student t-test was used to test the statistical significance. Data are expressed as the mean ± SE of 3 replicates. (Scale bars 200 μm, 100 μm). * p<0.05.

CCD-18Co fibroblasts seeded in type I collagen matrix were viable and showed steady growth over 5 days under mono- (data not shown) and co-culture conditions (S1 Fig). When fibroblasts were co-cultured with HT-29 TS for 6 days, prominent changes were observed in cell morphology and α-SMA expression levels: upon co-culture with cancer cells, fibroblasts showed signs of activation including increased levels of α-SMA and F-actin expression and F-actin stress fiber rendering the elongated spindle shape of fibroblasts (Fig 5A). Fibroblast activation was also indicated by an increase in migratory ability from the designated channel toward the tumor compartment (Fig 5B). Some fibroblasts migrated fast, crossing the whole 1 mm gap distance between the two channels and reached near to the tumor channel within 6 days.

### Differential drug sensitivity of HT-29 tumor spheroids induced by fibroblast co-culturing

**Fig 6:**
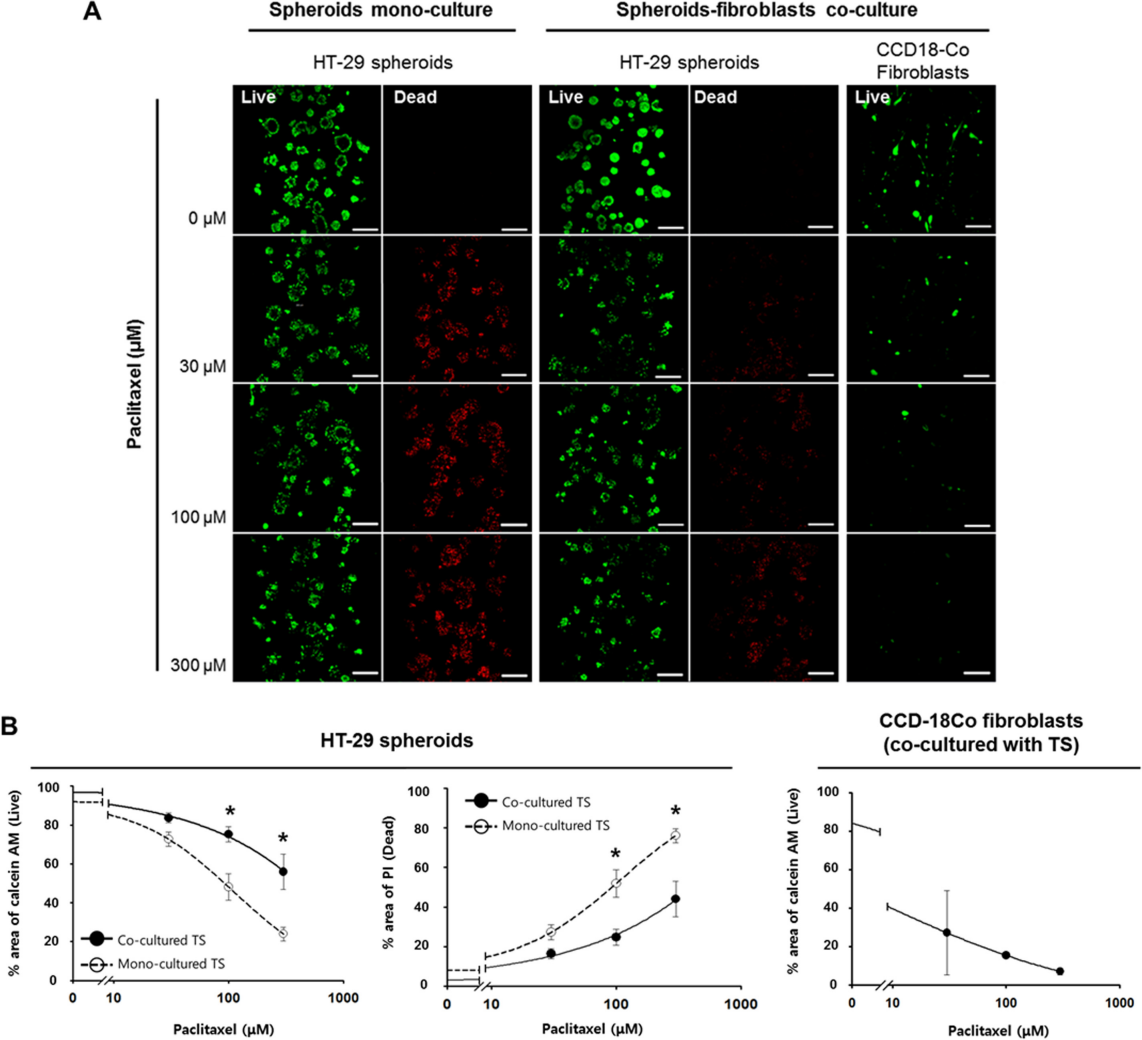
Differential sensitivity of HT-29 tumor spheroids to paclitaxel treatment. (A) Fluorescence images of HT-29 tumor spheroids and CCD-18Co fibroblasts stained with calcein AM (viable) and PI (dead). (B) Changes in the faction of viable and dead cells in HT-29 tumor spheroids and CCD-18Co fibroblast. Cells were grown for 5 days and stained for viability (calcein AM / PI) after 72 hr exposure to paclitaxel up to 300 μM under mono- or co-culture condition. Optical sections were acquired at 3 μm intervals and stacked into a z-projection. Images were quantified using image J software as described above. Student t-test was used to test the statistical significance. Data are expressed as the mean ± SE of 3 replicates. (Scale bar: 200 μm). * p<0.05.

Sensitivity to paclitaxel in HT-29 cells grown as TSs with or without co-culturing with CCD-18Co fibroblasts was compared. After 5 days of culture, TSs were exposed to paclitaxel-containing medium for 72 hr and changes in viability (calcein AM area as viable fraction; PI area as dead fraction) were determined (Fig 6A). Changes in the viability of TS were proportional to the drug concentration in both mono- and co-cultured TS, and a significantly reduced sensitivity was observed in the TS co-cultured with fibroblasts (Fig 6B). It was noteworthy that, in cancer-fibroblast co-culture, fibroblasts showed 80% cell kill at 30 μM, the lowest drug concentration tested and almost no viable cells were detected at 300 μM, indicating prominent sensitivity of fibroblast to paclitaxel as compared to tumor cells

### Differential protein expression in tumor spheroids upon co-culture with fibroblasts

**Fig 7:**
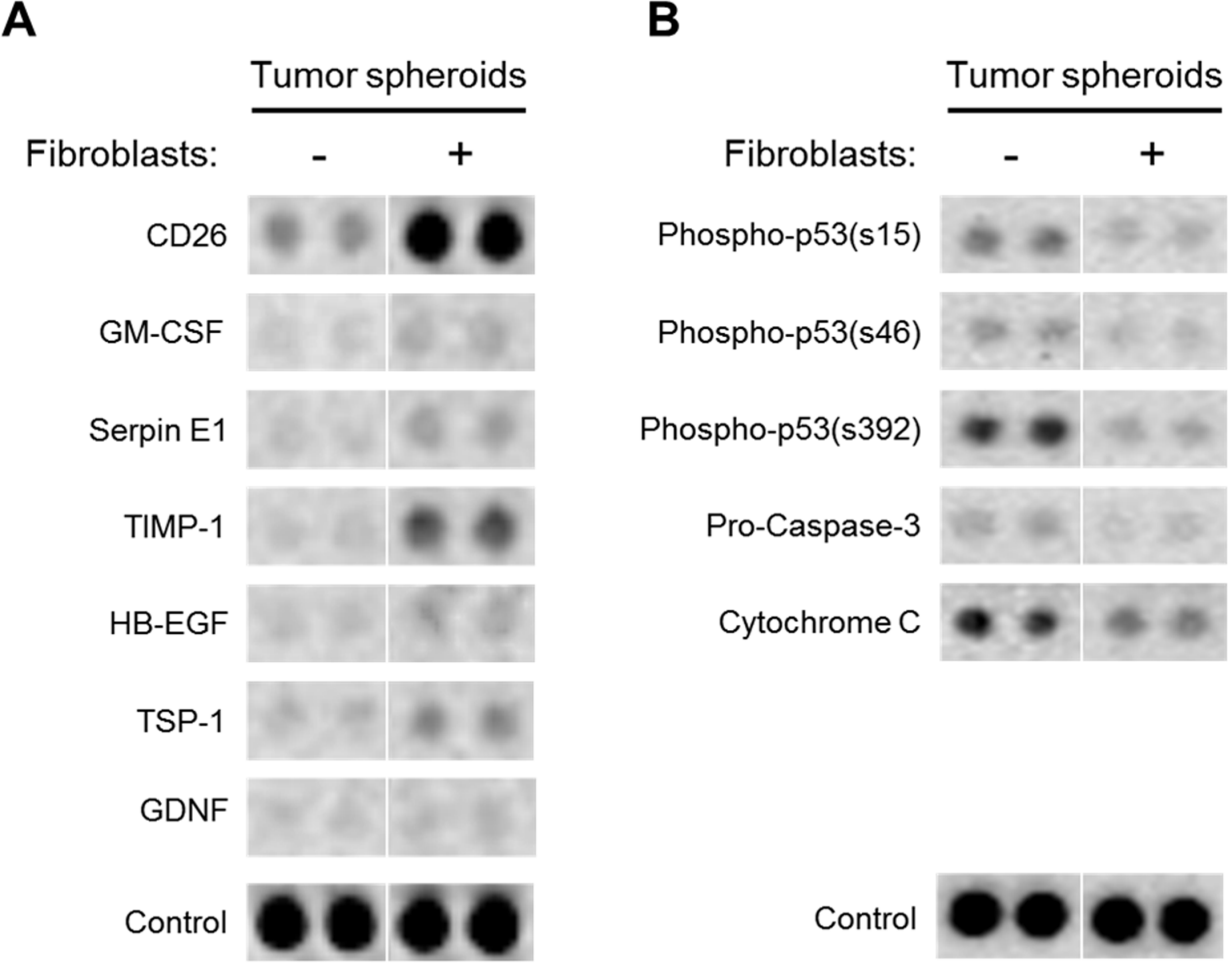
Differential protein expressions in HT-29 tumor spheroids with or without fibroblast co-cultures. When co-cultured with fibroblasts, TS showed up-regulation of 7 angiogenesis-related proteins with 1.5-fold or greater changes (A) and down-regulation of 5 apoptosis-related proteins with greater than 30% changes (B). HT-29 TSs were grown for 6 days with or without fibroblasts in microfluidic channels and harvested for analysis using Proteome Profiler™ (see Materials and Methods for details).

As angiogenesis and apoptosis are key events in tumor progression, we examined the changes in expression of the intracellular proteins related with these events in HT-29 TSs cultured for 6 days with or without fibroblast co-cultures. While 14 proteins out of 28 proteins related with angiogenesis -related showed upregulation in co-cultured TS, 7 factors were found to be significantly up-regulated with changes exceeding 1.5-fold e.g., CD26, GM-CSF, SerpinE1, TIMP-1, HB-EGF, TSP-1, GDNF (Fig 7A). Among apoptosis-related 35 different proteins, 5 factors were down-regulated with significant changes (≥ 30%) in HT-29 TSs co-cultured with fibroblasts, e.g., phospho-p53 (S15), phospho-p53 (S46), phospho-p53 (S392), Pro-caspase-3, Cytochrome C). None of the apoptosis-related proteins showed up-regulation in co-cultured TS compared to mono-cultured TS (Fig 7B).

## Discussion

In this study, we developed an *in vitro* colorectal 3D co-culture model using a microfluidic chip to mimic tumor-stroma interaction within microenvironment. We designed an indirect co-culture microfluidic chip without direct tumor cell-stromal cell contact, in which soluble factors may be transported across the medium channel (1000 μm) and potential development of local concentration gradients may occur (Fig 1). It is also noted that we used intermittent feeding instead of constant flow; there was no significant mixing effect between channels except the time of medium changes. The transwell system has been commonly used for indirect co-culture model studying anti-cancer drug resistance, angiogenesis, epithelial-mesenchymal transition (EMT) [32,34–35]. However, a critical drawback of the transwell system is a non-physiological distance between two cellular compartment and extensive dilution of soluble factors secreted into a large volume of medium. Lower concentrations are not able to exert the full effect of soluble factors, limiting the ability of the transwell model to mimic the bidirectional interaction within the microenvironment and resulting phenotypes *in vivo* (e.g., EMT and drug resistance) [32]. For example, an indirect transwell co-culture system between non-small cell lung cancer (NSCLC) and CAF failed to show EMT, unlike a direct co-culture system [35]. In contrast, a microfluidic chip can mimic a mutual or real-time interaction between cell populations under study and, therefore, is considered a better model when compared to the transwell model or conditioned-media method [36]. Meanwhile, mixed co-culture systems have also been utilized to study interactions among different type of cells within a tumor microenvironment [37,38]. Although these co-culture systems represent a more realistic pathophysiological model of a tumor, cell-type specific analysis is an important issue to overcome. In a study using a mixed co-culture of osteoblasts and endothelial cells, only limited information was obtained (e.g., phenotypic stability of endothelial cells and its formation of microvessel-like structures); no information on each cell population independently was captured [35]. When a simple, live labeling technique was used in a mixed co-culture model of two different cell types (i.e., human adult osteoblast cells and abdominal fibroblasts), information gleaned was limited to proliferation and spatial distribution of respective cell types [38]. By using PDMS replica-bonded to a film instead of glass in our microfluidic chip system, each cell population can be harvested and analyzed for gene expression [39]. Overall, our microfluidic chip system can be used effectively to study phenotypic changes resulting from reciprocal interaction between different type cells via soluble factors and corresponding changes in gene expression.

From day 2 after co-culture with TSs, CCD-18Co fibroblasts assumed a spindle shape with contracted stress fibers in the cytoplasm instead of squamous cell type of mono-cultured fibroblasts (data not shown). These morphological changes also coincided with the increased expression of α-SMA (Fig 5A). Prominent roles of fibroblasts in the growth and progression of cancers are well reported [19]. They are usually activated to be involved in the pathologic process such as cancer, hence the nature of this interaction is considered reciprocal. We observed changes in α-SMA expression of fibroblasts when co-cultured with TSs. Activated fibroblasts are commonly identified by mesenchymal markers such as fibroblast specific protein 1 (S10A4, FSP1), desmin, vimentin, paladin, urokinase-type plasminogen activator receptor associated protein (UPARAP), galectin-3, podoplanin, platelet derived growth factor receptor (PDGFR), and/or α-SMA [40]. Although other markers listed above and fibroblast activation protein (FAP) can be used to evaluate fibroblast activation, α-SMA is the most commonly used and reliable marker for the maturation of fibrocytes as it is a hallmark of fibroblast differentiation to myofibroblast [41]. These data support the activation of CCD-18Co fibroblasts co-cultured with TS in our model system.

Along with morphological changes, migration of activated fibroblasts towards tumor spheroids was observed at day 3 (S1 Fig). Fibroblasts, which moved out from their own channel, assumed a sharp and elongated morphology with polarity toward the leading edge (Fig 5). Migration of fibroblasts out of their channel was significantly increased towards tumor-loaded channel compared to non-tumor side (Fig 3B). This can be associated with a local accumulation and gradient of soluble factors resulting in differential condition among channels in our microfluidic chip as described above. Chemokines and growth factors and their receptors are known to mediate chemotaxis of cancer cells and cancer-associated stromal and inflammatory cells, e.g., CCL19- and CCL21-CCR7 and FGF-FGFR [42]. In addition to their roles in the growth and survival of cancer and stromal cells, the cascades of downstream signaling can lead to alterations in cytoskeletal dynamics resulting in chemotaxis [42]. It would be important and interesting to evaluate differential gene expression profile of these activated fibroblasts before and after acquisition of migratory ability, which may provide a potential target to inhibit interaction between tumor-stroma in addition to molecular mechanism involved in this process.

Co-culture-derived changes were also observed in TS, i.e., average size of co-cultured TSs was larger than mono-cultured TSs (Fig 3). As demonstrated by the size distribution at day 5, significantly increased fraction of spheroids was found as ‘large spheroids’ under co-culture conditions (Fig 3; S2 Fig). On the other hand, expression of a cell proliferation marker, Ki-67, decreased in co-cultured TSs grown for 6 days (Fig 4A). Decreased proliferation appeared to be related with EMT stimulation by fibroblast co-culture. In fact, a proliferation-inhibiting effect and a tumor-suppressive activity of CAF have been reported in relation to EMT promoting effect [19, 43]. By day 6, TS cultured with fibroblasts showed an additional EMT sign of an increased level of fibronectin (Fig 4B) as well as cell migration out of tumor spheroids (data not shown). Among ECM components, fibronectin overexpression has been associated with a poor outcome in gallbladder cancer patients [44] and EMT progression and cell migration ability *in vitro* [45]. It is noted that up-regulation of fibronectin coincided with the reduced uptake of DOX at higher concentration (300 μM) in co-cultured TSs (Fig 4C). These results are in agreement with previous reports in which increased stiffness caused by increased ECM deposition in tumor tissue was suggested as a physical barrier preventing intratumoral drug penetration [46]. Collectively, our model may serve as a useful model in investigating CAF-induced and ECM-related drug resistance as well as EMT in 3D tumors.

The anti-proliferative activity of paclitaxel showed a significant reduction in HT-29 TS co-cultured with fibroblasts as shown by calcein AM and propidium iodide staining (Fig 6). The decreased sensitivity of co-cultured TS may be associated with the changes related with EMT as shown by reduced Ki-67 expression and increased fibronectin expression in TS (Fig 4A and 4B) [43]. Many researchers have suggested that fibroblasts are key players in tumor microenvironment-mediated drug resistance [18, 47–48]. Emerging studies show an important role for by CAF-expressed growth factors in modulating drug resistance. Among these CAF-secreted growth factors, HGF signaling has been shown to be involved in drug resistance through the upregulation of MAK and AKT pathways [13] and in expansion of the cancer stem cell (CSC) population [49]. DOX resistance in breast cancer cells was shown to be associated with fibroblast-induced high mobility group box 1 (HMGB1) expression [50]. As shown with paclitaxel, our microfluidic chip will be a useful model to study drug resistance and its association with fibroblast-secreted factors.

It is well known that tumor progression is regulated by multiple modes of cancer-stroma interactions via soluble as well as contact components resulting in expression changes in protein involved in angiogenesis and apoptosis mechanisms [51]. We analyzed expression profiles of 63 proteins known for their roles in human angiogenesis and apoptosis induction in HT-29 TSs cultured with or without fibroblasts (Fig 7). Among the proteins shown a significant increase upon fibroblast-co-culture is CD26 protein (4.5-fold, Fig 7), also known as dipeptidyl-peptidase IV (DPPIV) and a serine peptidase associated with signal transduction, immune regulation and apoptosis [52]. Its association with some characteristics of CSCs has recently been reported, such as sphere formation *in vitro* and tumor development, metastasis and chemoresistance [53, 54]. It is also involved not only in cell-cell but also in cell-ECM interactions via an interaction with collagen and fibronectin [55]. In addition, significant increases were observed for GM-CSF (1.8-fold), Timp-1 (8.6-fold) Serpin E1, and tumor growth inducer HB-EGF (2.3-fold), TSP-1 (2.3-fold), GDNF (1.5-fold) in HT-29 TSs cultured with fibroblasts. These are known for factors associated with tumor progression [56–61]. Changes in Timp-1 and Serpin E1 were similar to the changes reported in our previous study in which co-culture media was analyzed. Along with upregulation of these proteins related with angiogenesis, apoptosis-related proteins, phosphorylated forms of p53 (ser15, Ser46, Ser392), and caspase 9-activating cytochrome C showed significantly decreased levels by 40% and 30%, respectively, in TS upon fibroblast co-culture. These results represent that decrease of apoptosis was induced by fibroblasts. Therefore, co-culture with fibroblasts induced changes of cellular proteins in TS towards tumor progression. Overall these data suggest that 3D architecture in tumor spheroids and the condition of proximity co-culture with fibroblasts allowing simultaneous mutual interaction among cancer cells and fibroblasts may increase the level of *in vivo* resemblance of our co-culture model.

Overall in the present study, we demonstrated that human cancer cells and fibroblasts can be co-cultured in 3D structures (1 mm-proximity) using a microfluidic chip, mimicking mutual microenvironmental interactions. The 3D interaction between these two cell types was confirmed by reciprocal activation, i.e., growth rate, ECM expression, drug accumulation, and drug sensitivity in TS, or by α-SMA expression, morphology changes and increased migration in fibroblasts. This 3D co-culture model can be useful as a tool to further study tumor microenvironment factors involved in the EMT process, fibroblast activation, and drug resistance.

## Supporting Information

S1 Fig Increased migration of fibroblasts activated by TS co-culture. Number of fibroblasts found outside of channels increased with time (▲arrow). (Scale bar: 200 μm).
(TIF)

S2 Fig Fibroblast activation induced TS size increase. Size distribution of tumor spheroids over 5 days of culture. Changes in size distribution were observed with longer incubation time. Size distribution pattern showed difference between mono-and co-cultured TSs.
(TIF)

S3 Fig The 63 antibodies of Human proteome array analysis. The 28 antibodies from angiogenesis array and 35 antibodies from apoptosis array were used for analysis.
(TIF)
