# Supplementary figures and images for: Co-Culture of Tumor Spheroids and Fibroblasts in a Collagen Matrix-Incorporated Microfluidic Chip Mimics Reciprocal Activation in Solid Tumor Microenvironment
Source: PLoS One. 2016 Jul 8;11(7):e0159013. doi: 10.1371/journal.pone.0159013 (PMC4938568; doi:10.1371/journal.pone.0159013)

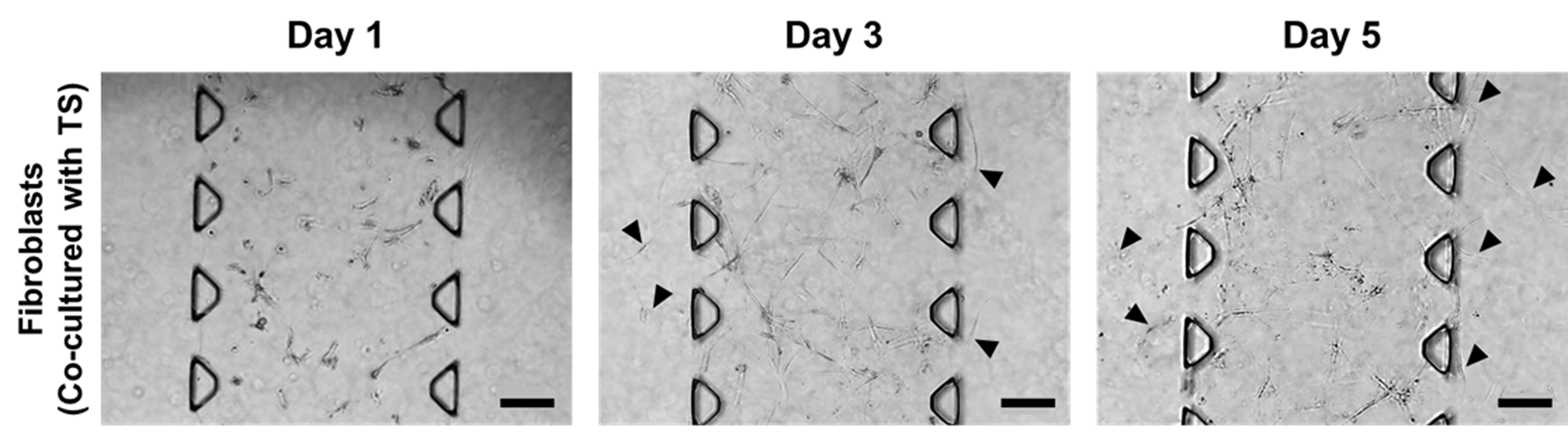

Supplement: S1 Fig — Number of fibroblasts found outside of channels increased with time (▲arrow). (Scale bar: 200 μm). (TIF) [file pone.0159013.s001.tif]

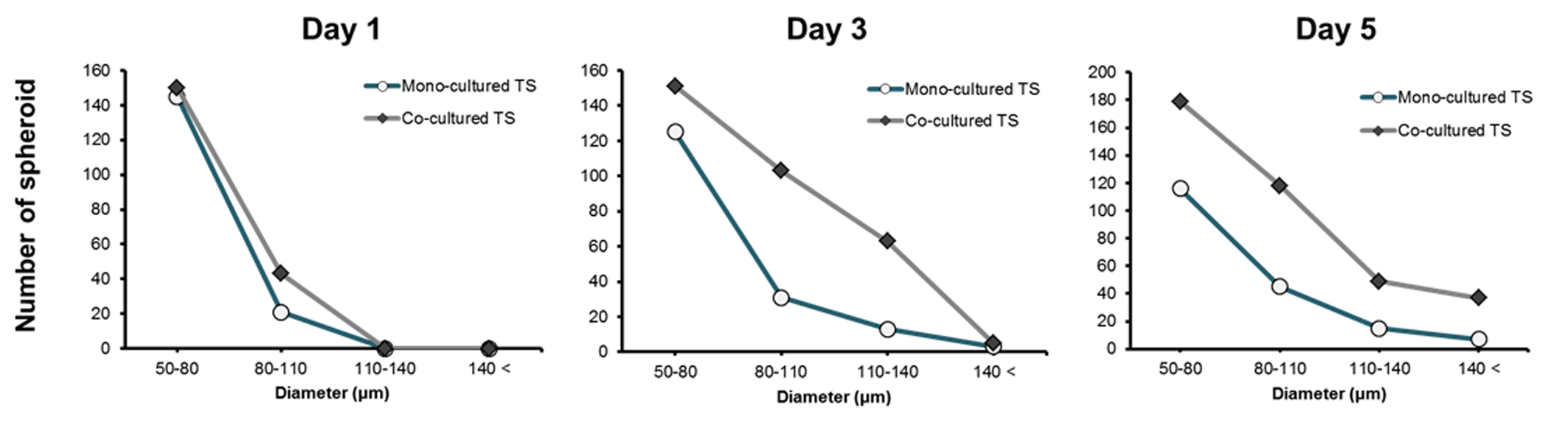

Supplement: S2 Fig — Size distribution of tumor spheroids over 5 days of culture. Changes in size distribution were observed with longer incubation time. Size distribution pattern showed difference between mono-and co-cultured TSs. (TIF) [file pone.0159013.s002.tif]

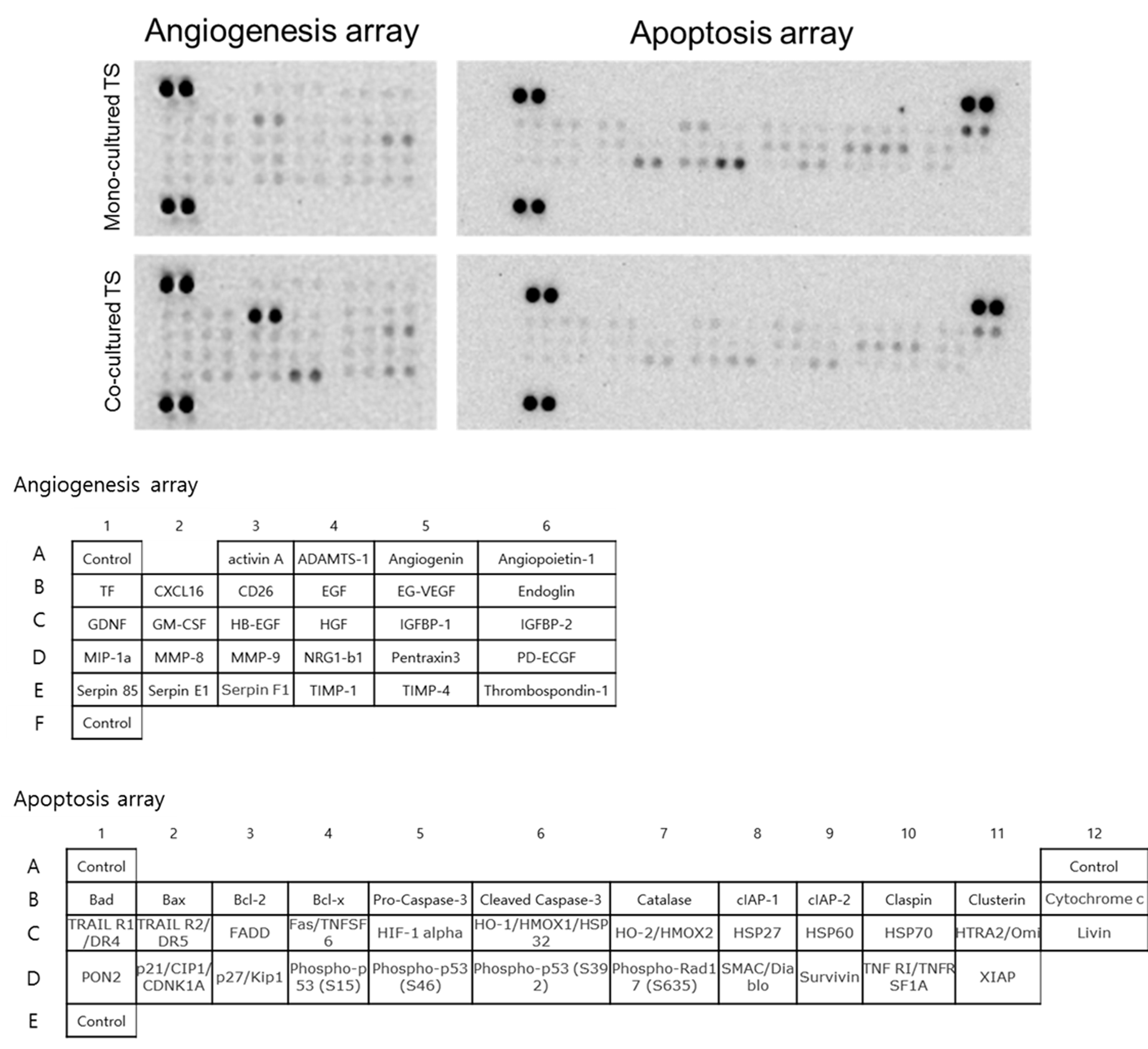

Supplement: S3 Fig — The 28 antibodies from angiogenesis array and 35 antibodies from apoptosis array were used for analysis. (TIF) [file pone.0159013.s003.tif]
